# The gut microbiota in experimental abdominal aortic aneurysm

**DOI:** 10.3389/fcvm.2023.1051648

**Published:** 2023-02-22

**Authors:** Jie Xiao, Zhanjie Wei, Chuanlei Yang, Shilin Dai, Xiancan Wang, Yuqiang Shang

**Affiliations:** ^1^Department of Cardiovascular Surgery, Central Hospital of Wuhan, Huazhong University of Science and Technology, Wuhan, China; ^2^Department of General Surgery, Central Hospital of Wuhan, Huazhong University of Science and Technology, Wuhan, China

**Keywords:** abdominal aortic aneurysm, gut microbiota, *Akkermansia*, *Bacteroidetes*, 16S rRNA

## Abstract

**Background:**

Abdominal aortic aneurysm (AAA) is a life-threatening disease and there are no effective treatments to inhibit aneurysm progression and rupture. The gut microbiota has been increasingly recognized, as a new therapeutic target, because of its role in host homeostasis. However, the role of the gut microbiota in AAA has not been clarified. Therefore, we performed 16S rRNA analysis to determine and compare the composition of the gut microbiota between AAA and control groups.

**Methods:**

We used the classical angiotensin-II induced AAA mouse model to investigate the role of gut microbiota and abdominal aortic aneurysm. The mice were randomly assigned to 2 groups: the control (*n* = 7) group received saline (vehicle), while the AAA (*n* = 13) group received solutions of Ang II. Aortic tissue and fecal samples were harvested 28 days after infusion. Fecal samples were analyzed by 16S rRNA sequencing.

**Results:**

The levels of *Oscillospira, Coprococcus, Faecalibacterium prausnitzii, Alistipes massiliensis*, and *Ruminococcus gnavus* were increased in the AAA group, while those of *Akkermansia muciniphila, Allobaculum*, and *Barnesiella intestinihominis* were increased in the control group. Furthermore, network analysis and ZiPi score assessment highlighted species in the phylum *Bacteroidetes* as the keystone species. PICRUSt2 analysis revealed that PWY-6629 (a super pathway of L-tryptophan biosynthesis), PWY-7446 (sulfoglycolysis), and PWY-6165 [chorismate biosynthesis II (archaea)] may-be involved in the metabolic pathways that contribute to AAA formation, and *E. coli*/*Shigella* may be the key bacteria that influence those three pathways.

**Conclusion:**

Alterations in the gut microbiota may be associated with the formation of AAA. *Akkermansia* and *Lactobacillus* were significantly decreased in the AAA group, but the keystone species in the phylum *Bacteroidetes* and the metabolic products of these bacteria should be given more attention in AAA formation research.

## 1. Introduction

Multiple compelling pieces of evidence have demonstrated that the composition and function of the gut microbiota can alter host homeostasis and result in obesity, inflammation, cardiovascular disease, and other diseases (1, 2). The gut microbiota is regarded as a human organ since it forms a complex community of interacting organisms and communicates with distal host organs through microbial metabolites (3). Recently, Tian et al. (4) reported that gut microbiota dysfunction plays key roles in the formation of abdominal aortic aneurysm (AAA). They found that *Roseburia intestinalis* and its metabolite butyrate significantly reduce neutrophil infiltration and the formation of NOX2-dependent neutrophil network structures, thereby reducing inflammation and promoting the formation of AAA. Moreover, Shinohara and his teammate (5) found that depleting the gut microbiota by oral antibiotic treatment can suppress macrophage accumulation and alleviate AAA development. These results have highlighted the role of the gut microbiota in the development of AAA.

AAA usually occurs in the infrarenal part of the aorta and is usually described as a weakening and dilatation of the abdominal aorta (6). AAA is usually asymptomatic unless complications occur; such complications lead to 150,000–200,000 deaths each year worldwide (7). Either open operation or endovascular repair is an effective method for treating patients with large, asymptomatic AAAs or symptomatic or ruptured AAAs of any size (8). However, few effective non-invasive therapy strategies can prevent the progression of abdominal aortic aneurysms.

Herein, we provide new insights into the gut microbiota associated with AAA in an Ang II-induced experimental abdominal aortic aneurysm (EAAA) mouse model to elucidate alterations in the composition of the gut microbiota and the potential mechanisms of action related to AAA formation.

## 2. Materials and methods

### 2.1. Ang II-induced EAAA model

All animal studies were approved by the Ethical Approval for Formation Review of Experimental Animal Welfare and Ethics, Zhongnan Hospital of Wuhan University (ZN2022173). A total of 20 male apolipoprotein E-deficient C57BL/6 mice (ApoE^–/–)^ aged 12 weeks and weighing 28–30 g were obtained from Beijing HFK Bioscience Co., Ltd. (HFK). All mice were housed under environmentally controlled specific pathogen-free conditions with a 12:12-h light-dark cycle and fed standard laboratory chow and tap water *ad libitum*. Ang II (Sigma-Aldrich; 1,000 ng/min/kg) or saline was administered to these ApoE^–/–^ mice through an osmotic mini-pump (model 2004, ALZET Osmotic Pumps) for up to 4 weeks. The mice were randomly assigned to 2 groups: the control (*n* = 7) group received saline (vehicle), while the AAA (*n* = 13) group received solutions of Ang II. Aortic tissue and fecal samples were harvested 28 days after infusion. Sample specimens were immediately flash frozen and subsequently stored at −80°C.

### 2.2. Assessment of EAAA

A high-resolution Vevo 2100 microimaging system (Visualsonic) was used to measure the aortic diameter in each group of mice on days 0 and 28. A suprarenal aortic diameter increase of ≥50% or the occurrence of aortic dissection (AD) in the mice was considered aneurysmal. Furthermore, the survival ratios were monitored daily, and a Softron BP-2010 Series system (a non-invasive tail-cuff system) was used to measure the systolic blood pressure on days 0 and 28.

### 2.3. DNA extraction and 16S rRNA gene-based analysis

Bacterial DNA extraction and sequencing of the 16S rRNA gene were conducted by Personalbio Technology Co., Ltd. (Shanghai, China). Briefly, fecal samples of approximately 200 mg per mouse were collected and used for DNA extraction with a QIAamp DNA stool Mini Kit (QIAGEN, Hilden, Germany) according to the manufacturer’s protocol. A NanoDrop 2000 (Thermo Scientific) was used to evaluate the DNA concentration and purity. The V3-V4 hypervariable regions of the 16S rRNA gene were amplified from the DNA extracts with primers (forward primer: ACTCCTACGGGAGGCAGCA and reverse primer: GGACTACHVGGGTWTCTAAT). The samples were sequenced on an Illumina NovaSeq6000 PE 250 system to obtain raw data. QIIME2 and DADA2 were used to denoise the raw data and obtain clean amplicon sequence variants (ASVs)/operational taxonomic units (OTUs). Each ASV/OTU sequence was annotated using QIIME2 (version 2019.4). R software (version 4.0.2) was used to draw taxonomic trees in packed circles (R ggraph, ggplot2 package). Krona software provides an interactive display of the taxonomic composition of the community. QIIME2 (version 2019.4) was used to calculate the alpha diversity index (e.g., Chao1 and Shannon diversity index) and beta-diversity. R software (version 4.0.2) was used to draw the rank-abundance curves (R ggplot2 package), to carry out principal coordinate analysis (PCoA) and non-metric multidimensional scaling (NMDS) based on the Bray-Curtis distance (R ape package and vegan package), to generate the heatmap of genus abundance (R pheatmap package), and to compare the sample groups in pairs with the MetagenomeSeq method (R metagenomeSeq package). Furthermore, linear discriminant analysis (LDA) effect size (LefSe) (9) and random forest analysis (QIIME2, version 2019.4) were used to analyze the difference between the control and AAA groups. Phylogenetic investigation of communities by reconstruction of unobserved states (PICRUST2) (10) was used to predict the function of the gut microbiota. Network analysis (11) was used to determine the key species based on the composition distribution of species in each sample. Moreover, network connectivity based on the ZiPi score is often used to indicate keystone species (12). The ZiPi score was calculated with R software (version 4.0.2) using the igraph package. R was used to calculate the Zi and Pi score values of each node in the network according to the modular cutting results of the co-occurring network. The Zi value refers to within-module connectivity, while the Pi value refers to among-module connectivity. Then, the role of each node in the associated network was determined according to the Zi and Pi score values. Nodes in a network (ASV/OTU) can be divided into four parts using Zi and Pi values, which are peripherals, connectors, module hubs and network hubs. In ecological research, peripherals represent specialists in microbial networks, module hubs and connectors primarily represent species that are close to generalists, and network hubs represent supergeneralists in microbial networks.

### 2.4. Statistical analysis

All data are presented as the means ± SEMs, and a *P* value <0.05 was considered to indicate statistical significance. The χ^2^ test was used to analyze the incidence of AAA. Two-tailed Student’s t test (for parametric data) was used to analyze differences between two groups. In addition, one-way analysis of variance (ANOVA) with the Bonferroni correction was used to compare multiple groups of Ang II/ApoE mice. Furthermore, an adjusted *P* < 0.05 was deemed to be significant depending on specific cases. The statistical analyses were performed using Prism 5.0 (GraphPad Software, La Jolla, CA).

## 3. Results

### 3.1. Ang II-induced experimental abdominal aortic mouse model

**FIGURE 1:**
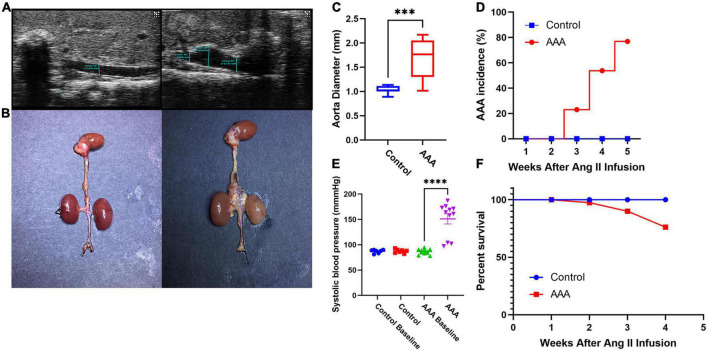
Ang II-induced experimental abdominal aortic aneurysm mouse model. **(A)** Typical ultrasound images of the abdominal aortic aneurysm from each group 28 days after infusion. **(B)** Representative photographs showing the visible changes in each group. **(C)** Aortic diameter (AAA group *vs.* control group, 1.703 ± 0.40 mm vs. 1.03 ± 0.08 mm, *P* < 0.05). **(D)** AAA incidence (AAA group: 2 died from AD on days 8 and 17, control group: 0, *P* < 0.05). **(E)** Systolic blood pressure (systolic blood pressure of mice in the AAA group on day 28 was significantly higher than that at baseline on day 0, *P* < 0.05). **(F)** Survival percentage (AAA group: 2 died from AD on days 8 and 17, control group: 0, ***represent *P* < 0.01, ****represent *P* < 0.001).

After 28 days of perfusion, 10 of 13 mice (76.9%, Figure 1D) in the AAA group were diagnosed with AAA (2 died from AD on days 8 and 17), while none of the mice in the control group were diagnosed with AAA (*P* < 0.05) (Figures 1A, B). In addition, the aortic diameter in the AAA group was significantly larger than that in the control group (1.703 ± 0.40 mm vs. 1.03 ± 0.08 mm, *P* < 0.05, Figure 1C), and the systolic blood pressure of mice in the AAA group on day 28 was significantly higher than that at baseline (on day 0, *P* < 0.05, Figure 1E). Furthermore, the survival ratio (Figure 1F) was notably higher in the control group (100%) than in the AAA group (84.6%, 2 died from AD on days 8 and 17).

### 3.2. Comparison of the gut microbiota between the AAA and control groups

**FIGURE 2:**
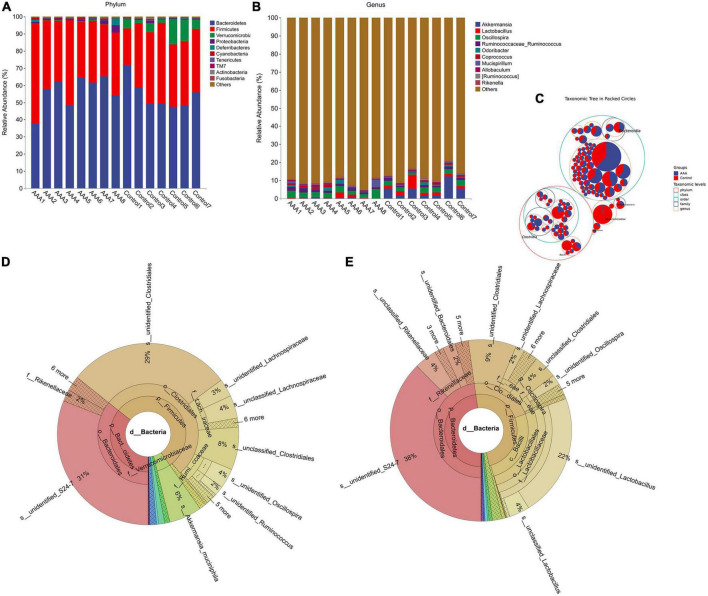
16S rRNA sequencing revealed alterations in the gut microbiota between the AAA and control groups. **(A)** Phylum level; **(B)** genus level; **(C)** taxonomic tree in packed circle; **(D,E)** Krona analysis of the bacterial community structures of the AAA and control groups.

After bioinformatic analysis was performed with QIIME2, we found that *Bacteroidetes, Firmicutes, Verrucomicrobia*, and *Proteobacteria* were the dominant phyla in both the AAA group and the control group (Figures 2A, C). However, the genera *Akkermansia* and *Lactobacillus*, which are always regarded as beneficial bacteria in human beings, were significantly decreased in the AAA group (Figures 2B–E).

### 3.3. The alpha and beta diversities of the gut microbiota

**FIGURE 3:**
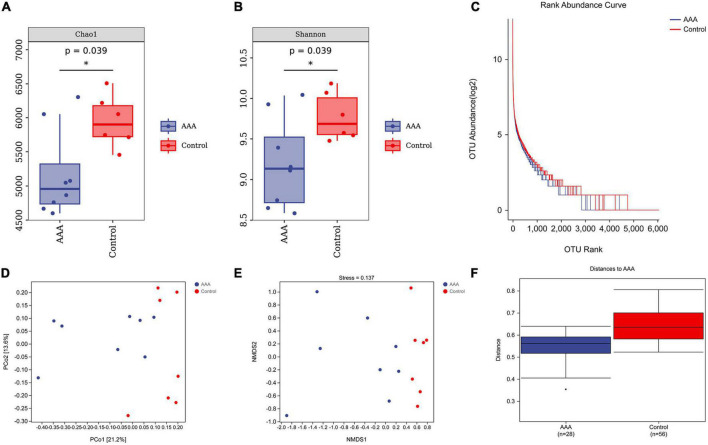
Alpha and beta diversities of the microbiota. QIIME2 (version 2019.4) was used to calculate the alpha diversity index and beta-diversity. **(A)** Chao1 estimator; **(B)** Shannon diversity index; **(C)** rank-abundance Curve; **(D)** PCoA analysis; **(E)** NMDS analysis, stress value, 0.137; **(F)** ANOSIM analysis, *R* = 0.32, *represent *P* < 0.01.

The Chao1 estimator and Shannon diversity index were used to calculate the richness and diversity of the gut microbiota, respectively (Figures 3A, B). In addition, we drew a rank-abundance curve to verify the richness and evenness of the gut microbiota (Figure 3C). These results demonstrated the low alpha diversity of the AAA group compared with that of the control group (*P* < 0.05).

Moreover, beta diversity was calculated by PCoA and non-metric multidimensional scaling (NMDS) analysis (Figures 3D, E). The stress value of NMDS was 0.137, which indicates the reliability of the NMDS analysis. The ANOSIM analysis demonstrated that the differences between groups were greater than the differences within groups (*R* = 0.32, *P* < 0.01, Figure 3F).

Taken together, these results demonstrated that the gut microbiota may play an essential role in the progression of AAA.

### 3.4. Species differences and marker species analysis

**FIGURE 4:**
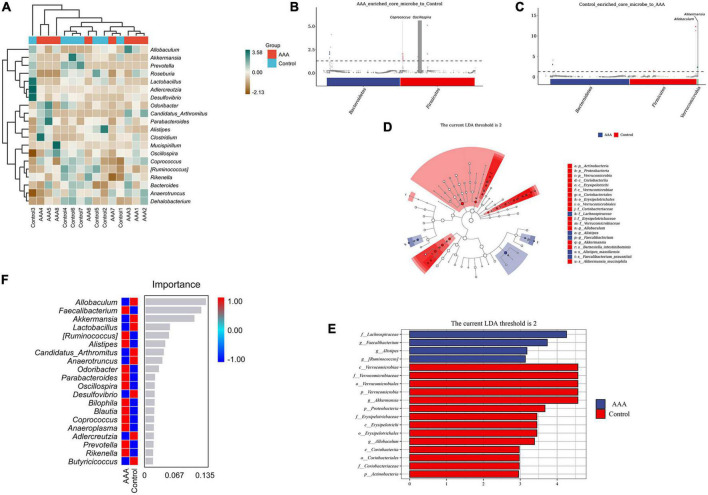
Species differences and marker species analysis. **(A)** Species composition heatmap, red bar represents the control group, blue bar represents the AAA group; **(B,C)** MetagenomeSeq analysis, red bar represents *Bacteroidetes*, blue bar represents *Firmicutes*; **(D,E)** linear discriminant analysis (LDA) effect size (LEfSe) analysis was used to analyze the difference between the control and AAA groups, red bar represents control group, blue bar represents AAA group; and **(F)** random forest analysis, red color represents high abundance in each group, blue color represented low abundance in each group.

Having explored the differences in microbial community composition (i.e., beta diversity), we also needed to determine which species were primarily responsible for these differences. A species composition heatmap, metagenomeSeq analysis, LEfSe analysis (Linear discriminant analysis Effect Size), and random forest analysis were used to filter out the dominant species in the AAA and control groups. The heatmap demonstrated the differential expression of the intestinal microbes in each group (Figure 4A). In the MetagenomeSeq analysis, we found that the AAA group was enriched in *Oscillospira* and *Coprococcus* (Figure 4B), while the control group was enriched in *Akkermansia* and *Allobaculum* (Figure 4C). The LFfSe analysis demonstrated that *Faecalibacterium prausnitzii, Alistipes massiliensis*, and *Ruminococcus gnavus* were significantly increased in the AAA group, and that *Akkermansia muciniphila* and *Barnesiella intestinihominis* were increased in the control group (Figures 4D, E). The random forest analysis revealed the degree of importance of the genera *Allobaculum* and *Akkermansia* in the control group and the genus *Faecalibacterium* in the AAA group (Figure 4F).

### 3.5. Network analysis and ZiPi score to reveal the keystone species

**FIGURE 5:**
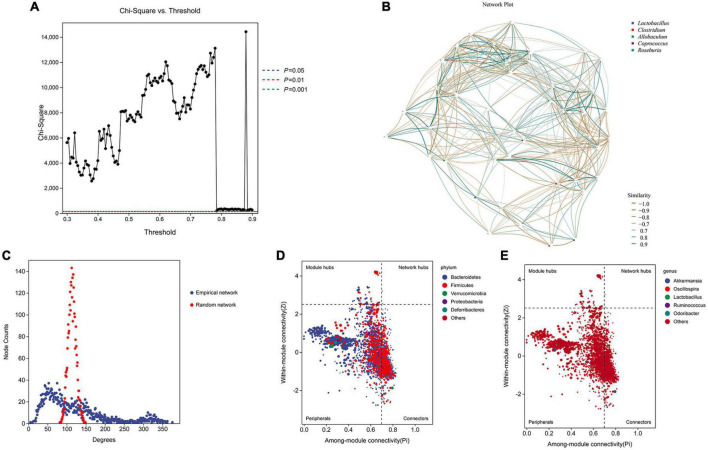
Network analysis and ZiPi score to determine the keystone species. **(A,B)** Network analysis was used to determine the key species based on the composition distribution of species in each sample; **(C)** topological index analysis; **(D,E)** ZiPi score was used to indicate keystone species.

We performed network analysis to identify the relationship between the microbiome and host environment. The network analysis demonstrated that the phyla *Firmicutes* and *Bacteroidetes* may play an essential role in influencing the host environment (Figures 5A, B). Topological index analysis revealed that the distribution of the gut microbiota fits a scale-free network, which means that the main factor influencing host homeostasis is the dominant microbiota (Figure 5C). Additionally, the ZiPi score verified that the keystone species that influenced host homeostasis belonged to the phylum *Bacteroidetes* (Figure 5D), however, the genus level was not identified (Figure 5E).

### 3.6. PICRUSt2 analysis to predict the potential role of the gut microbiota in AAA formation

**FIGURE 6:**
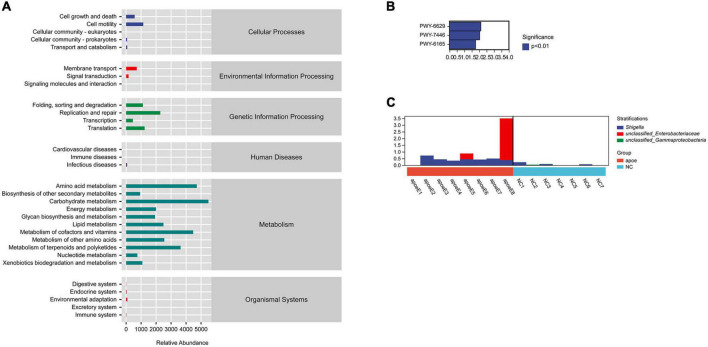
PICRUSt2 analysis to predict the potential role of the gut microbiota. **(A)** The gut microbiota participating in metabolic pathways; **(B)** different metabolic pathways between the AAA and control groups; **(C)** species composition analysis.

We used PICRUSt2 (Phylogenetic Investigation of Communities by Reconstruction of Unobserved States) to predict the potential role of the gut microbiota in AAA formation. The function of the gut microbiota predicted to play a primary role in host homeostasis was biosynthesis; other predicted functions included degradation, utilization, assimilation, detoxification, generation of precursor metabolites and energy, glycan pathways, macromolecule modification, metabolic clusters, and so on (Figure 6A). In addition, we found that the PWY-6629 (a super pathway of L-tryptophan biosynthesis), PWY-7446 (sulfoglycolysis), and PWY-6165 [chorismate biosynthesis II (archaea)] metabolic, pathways differed between the AAA and control groups (*P* < 0.05) (Figure 6B). Through species composition analysis, we found that *E. coli/Shigella* may be the key bacterial group that influences those three pathways (Figure 6C).

## 4. Discussion

The gut microbiota has gained recognition as a result of its role in homeostasis and is considered as a new treatment target for many diseases (13). In this study, we performed 16S rRNA analysis to identify alterations in the gut microbiota between the EAAA and control groups. We hypothesized that the gut microbiota may participate in the progression of abdominal aortic aneurysms. For example, different alpha and beta diversities revealed that the gut microbiota in the AAA and control groups differed.

The species composition map demonstrated that the genus levels of *Akkermansia* and *Lactobacillus* were significantly decreased in the AAA group compared with the control group. Furthermore, thorough investigations via MetagenomeSeq analysis, LEfSe analysis, and random forest analysis showed that the levels of *Oscillospira, Coprococcus, Faecalibacterium prausnitzii, Alistipes massiliensis*, and *Ruminococcus gnavus* were increased in the AAA group, while the levels of *Akkermansia muciniphila, Allobaculum*, and *Barnesiella intestinihominis* were increased in the control group. *Akkermansia* was first described in 2004 and many studies have been conducted to investigate its potential role in humans (14). Previous studies have demonstrated that *Akkermansia* is a promising target for treating intestinal microbiota-related diseases, such as colitis, metabolic syndrome, and immune diseases (15). Recently, He et al. (16) reported that *Akkermansia muciniphila* inhibited the formation of AAA by restoring gut microbiota diversity, and altered the expression of peripheral immune factors. *Barnesiella intestinihominis* is also regarded as a beneficial bacterium for host homeostasis. A study published in 2016 found that *Barnesiella intestinihominis* accumulated in the colon and promoted the infiltration of γδT cells to produce IFN-γ in cancer lesions and ultimately ameliorated the efficacy of cyclophosphamide (CTX) to inhibit the progression of cancer (17). However, *Oscillospira* may play a bidirectional role in host metabolism. Zha et al. revealed that *Oscillospira* was likely to drive the microbiome in patients with type 2 diabetes mellitus to a more dysbiotic status (18). Another study demonstrated that the increased abundance of the genus *Oscillospira* may exert protective actions by enhancing short-chain fatty acids, NAD + metabolism, and sirtuin activity to increase fatty acid oxidation and ultimately inhibit the progression of non-alcoholic steatohepatitis (19). Therefore, further studies are needed to clarify the mechanism of action of *Oscillospira* in AAA progression. Moreover, *Coprococcus* has been demonstrated to be positively related to obesity, and a recent study found that inulin can decrease the level of the genus *Coprococcus* and ameliorate the moods of obese patients (20). *Faecalibacterium prausnitzii* is usually regarded as a probiotic for human metabolism (21). However, Filippis et al. enrolled 120 children (90 children with allergies and 30 age-matched healthy controls) to investigate alterations in the gut microbiota and found a high abundance of *Faecalibacterium prausnitzii* in children with allergies (22). In the human *Faecalibacterium* complex, eleven clades have been identified (clade A through clade K) as *F. prausnitzii* (23). Filippis et al. (23) reported that *Faecalibacterium prausnitzii* is associated with a Westernized lifestyle, and a Western diet has a different prevalence of *Faecalibacterium* clades. For example, the prevalence of clade A and clade D was higher in a Western diet group, while the other clades were higher in a non-Western diet group. It is known that Western diet is associated with the formation of abdominal aortic aneurysm (24). In our study, we found that the abundance of *Faecalibacterium prausnitzii* was increased in the AAA group, and the potential explanation may be that *Faecalibacterium prausnitzii* clade A or clade D was detected. We believe this phenomenon is very interesting, and we will intend to clarify the role of *Faecalibacterium prausnitzii* in the formation of AAA in future studies. *Alistipes massiliensis* belongs to the *Alistipes* genus, which is a relatively newly identified genus of bacteria. There is a bidirectional function of *Alistipes* that may have protective effects against some diseases, such as cancer, colitis, and liver fibrosis. In contrast, other studies have indicated that *Alistipes* may participate in the formation of colorectal cancer and depression (25). *Ruminococcus gnavus* is an anaerobic, gram-positive bacterium that can digest intestinal mucus and produce an inflammatory substance. A recent cohort study found that *Ruminococcus gnavus* was directly associated with percent body fat and induced metabolic syndrome (26).

Although the studies above highlighted notable species of the gut microbiota, our network analysis and ZiPi score assessment demonstrated that the keystone species involved in AAA formation were those in the phylum *Bacteroidetes*. *Bacteroidetes* are gram-negative, anaerobic bacteria that are resident flora in humans. They have an outer membrane, a peptidoglycan layer, and a cytoplasmic membrane. Their main products include acetic acid, isovaleric acid, and succinic acid (27). The *Bacteroidetes* phylum may play a key role in host homeostasis, and its impact on host health and disease is complex, as it involves the catabolism of ingested complex polysaccharides, colonization resistance to pathogens, the production of B vitamins, and support for other anaerobic microorganisms (28). Recently, Yao et al. (29) found that the gut bacterial phylum *Bacteroidetes* which expresses selective bile salt hydrolase (BSH) activity, plays an essential role in maintaining host homeostasis. Germ-free mice colonized with hydrolase-deleted bacteria maintained a better weight and lower levels of fat in their blood and liver. Furthermore, those mice shifted to burning fat instead of carbohydrates for energy and regulated immune pathways in the gut. Therefore, the *Bacteroidetes* phylum may play an essential role in AAA formation, and further studies are needed for clarification.

In addition, PICRUSt2 analysis was conducted to predict the potential role of the gut microbiota. We found that PWY-6629 (a super pathway of L-tryptophan biosynthesis), PWY-7446 (sulfoglycolysis), and PWY-6165 [chorismate biosynthesis II (archaea)] may contribute to the different metabolic pathways between the AAA and control groups. Through species composition analysis, we found that *E. coli*/*Shigella* may be the key bacterial taxon that influences these three pathways. Future studies are needed to clarify the potential mechanism that influences AAA formation.

Some limitations should be considered. First, human samples are needed for sequence analysis. Although the Ang II-induced EAAA mouse model could imitate the pathology of human AAA, the gut microbiota of humans and mice are different. Second, shotgun metagenomic sequencing is better than 16S rRNA analysis, and more data should be obtained through shotgun metagenomic sequencing. Shotgun metagenomic sequencing is better for functional profiling, taxonomic resolution (bacterial species, sometimes strains and single nucleotide variants, if sequencing is deep enough), coverage of all taxa (including viruses), bioinformatics requirements, high sensitivity to host DNA contamination, and lower bias, but usually costs too much compared with 16S rRNA (30).

## 5. Conclusion

Alterations in the gut microbiota may be associated with the formation of abdominal aortic aneurysm. *Akkermansia* and *Lactobacillus* were significantly decreased in the AAA group, but the keystone species were found to belong to the phylum *Bacteroidetes*, and the metabolic products of these bacteria should be given more attention in AAA formation research.

## Data availability statement

The datasets presented in this study can be found in online repositories. The names of the repository/repositories and accession number(s) can be found below: https://dataview.ncbi.nlm.nih.gov/object/PRJNA890908?reviewer=6gvcn9j2pb4d7drg85e4jpjhb, PRJNA890908.

## Ethics statement

The animal study was reviewed and approved by Institutional Animal Care and Use Committee of Tongji Medical College, Huazhong University of Science and Technology.

## Author contributions

JX, ZW, and CY designed and performed the experiments, analyzed the data, and wrote the manuscript. YS reviewed the manuscript. SD and XW performed the experiments. All authors contributed to the article and approved the submitted version.
